# Factors Associated with HIV Prevalence and HIV Testing in Sierra Leone: Findings from the 2008 Demographic Health Survey

**DOI:** 10.1371/journal.pone.0137055

**Published:** 2015-10-09

**Authors:** Nataliya Brima, Fiona Burns, Ibidun Fakoya, Brima Kargbo, Suleiman Conteh, Andrew Copas

**Affiliations:** 1 Research Department of Infection and Population Health, University College London, London, United Kingdom; 2 Royal Free London NHS Foundation Trust, University College London, London, United Kingdom; 3 Ministry of Health and Sanitation, Freetown, Sierra Leone; 4 National AIDS Control Program, Freetown, Sierra Leone; International AIDS Vaccine Initiative, UNITED STATES

## Abstract

**Background:**

The Sierra Leone Demographic Health Survey 2008 found an HIV prevalence of 1.5%. This study investigates associations with HIV infection and HIV testing.

**Methods:**

Households were selected using stratified multi-stage sampling. In all selected households women aged 15–49 were eligible. In every second household men aged 15–59 were also eligible. Participants were asked to consent for anonymous HIV testing. All participants interviewed and tested were analysed. Multiple logistic regression identified associations with HIV infection, undiagnosed infection and with ever having a voluntary HIV test among sexually active participants.

**Results:**

Of 7495 invited 86% **(**6,475) agreed to an interview and HIV test. Among 96 HIV positive participants, 78% had never taken a voluntary HIV test so were unaware of their serostatus, and 86% were sexually active in the last 12 months among whom 96% did not use a condom at last intercourse. 11% of all participants had previously voluntarily tested. Among women who had tested, 60% did so in antenatal care. We found that those living in an urban area, and those previously married, were more likely to be HIV infected. Voluntary HIV testing was more common in those aged 25–44, living in an urban area, females, having secondary or higher education, having first sexual intercourse at age 17 years or older, and using condoms at last sex. Although 82% of men and 69% of women had heard of HIV, only 35% and 29% respectively had heard of antiretroviral therapy.

**Conclusions:**

The HIV prevalence in Sierra Leone has been stable. HIV testing, however, is uncommon and most infected individuals are unaware of their serostatus. This could allow the epidemic to escalate as individuals with undiagnosed infection are unlikely to change their behaviour or access treatment. Improving knowledge and increasing testing need to remain central to HIV prevention interventions in Sierra Leone.

## Introduction

Several studies across Africa have reported that up to 84% of infected individuals are unaware of their HIV status [1–4], the vast majority of whom remain sexually active [1,2,4].

Sexual behaviour [5–10] and factors that influence the transmission of HIV during sexual intercourse [11] (such as sexually transmitted infections (STIs) [12], male circumcision [13] and condom use [11]), are acknowledged as the main risk factors associated with the generalised HIV epidemic that has taken hold in sub-Saharan Africa. There is a substantial risk of onward sexual transmission from people living with HIV/AIDS (PLWHAs) who have not yet started antiretroviral therapy but continue to have unprotected sex [14,15].

HIV prevention for PLWHA alongside universal HIV testing has been recommended across Africa [16, 17]. Between 2003 and 2011, forty-seven Demographic Health Surveys (DHS) were conducted in 29 sub-Saharan countries [18]. While uptake of HIV testing has increased in every country studied [18], and standalone VCT sites and provider-initiated HIV-testing and counselling in health facilities has increased, the proportion of individuals aware of their HIV status remains low [16, 19–21]. Across surveys a large majority of women (71 percent) and men (83 percent) have never been tested for HIV [18].

Scaling up access and outreach to testing among underserved populations remains an important goal on the road to achieving universal access to treatment and support in most countries. Hence it is important for every country to understand associations with testing coverage and with infection so as to make effective plans to contain the epidemic.

Sierra Leone, a country in West Africa with an estimated population of 5.6 million, remains at the bottom of the list of all countries in terms of its development indicator [22]. Sierra Leone is divided into 4 regions-Northern, Eastern, Southern and Western. The capital Freetown is located in Western region. Sierra Leone has scaled up HIV Testing and Counselling (HTC) capacity since 2006 [23], including traditional voluntary HIV testing centres and mobile testing sites.

In Sierra Leone the HIV prevalence was reported at around 2% in the general population (aged 15–49) from two national sero-prevalence surveys conducted in 2002 and 2005. The Sierra Leone Demographic Health Survey (SLDHS) 2008 is the only sero-prevalence survey with data available to include all areas of Sierra Leone and also include an HIV testing component where the HIV results were anonymously linked with the broader behavioural, social, and demographic information collected. The HIV prevalence estimate for the general population (female participants aged 15–49 and male participants aged 15–59) from the SLDHS 2008 is 1.5% and it shows that the prevalence has stabilised and even possibly slightly decreased [24].

Very little has been published on the HIV epidemic within Sierra Leone [25,26], and the factors associated with HIV prevalence and HIV testing in the general population of Sierra Leone have not previously been explored in any depth. In this paper, through a more extensive analysis than conducted previously [24], we aim to identify factors associated with HIV infection and with having taken a voluntary HIV test using the SLDHS 2008.

## Methods

### Survey methodology

In 2008 Sierra Leone conducted its first SLDHS. The 2008 SLDHS sample was a stratified clustered sample selected in two stages from the 2004 census frame. Administratively, Sierra Leone was divided into 19 local councils. Strata were defined by separating each local council into its urban and rural areas. The allocation of a target sample size to each stratum was not proportional to stratum size and ‘over-sampled’ the smaller strata. Within each stratum census enumeration areas were sampled proportional to size and formed the primary sampling units for the survey. Within each selected enumeration area 22 households were selected by equal probability systematic sampling. Further details of the sampling are available elsewhere [24]. Within the selected households all women aged 15–49 who slept there the night before the survey were eligible to be interviewed. All men aged 15–59 who slept in half the selected households were eligible to be interviewed. Three types of questionnaires were used for the 2008 SLDHS: a household questionnaire, a women’s questionnaire and a men’s questionnaire. The household questionnaire included questions used to identify women and men eligible for individual interviews and to obtain voluntary consent for anonymous HIV testing. The men’s and women’s questionnaires included standard sets of socio-demographic questions, and the sexual activity and HIV/AIDS sections included questions on first sex, recent sexual activity (last 12 months), knowledge, attitudes, behaviour, stigma around HIV, testing and sexual health in the last 12 months[24]. For our analysis we have included data from all questions on first sex and recent sexual activity except for those relating to paid sex in the last 12 months as the reported prevalence was too low to be informative. We decided not to include information on the number of life time partners because these data were not provided by nearly a half of participants. We included data from all knowledge, testing and attitudes towards HIV questions. Participants were interviewed face-to-face.

### HIV Testing

All women and men who were eligible for the interview in households selected for the male survey in the SLDHS 2008 were asked to provide three drops of blood for HIV testing [24]. The protocol for the blood specimen collection and analysis was based on the anonymous linked protocol developed by the DHS programme and was reviewed and approved by the Sierra Leone National Ethics Committee on Bio-Medical Research. For the purposes of blood sample collection, to obtain informed consent for collecting blood for HIV testing, interviewers explained the procedures, the confidentiality of the data, the fact that test results could not be linked or made available to the subject, and informed respondents how they could establish their HIV status through voluntary counselling and testing (VCT) services. The HIV test results were merged with the individual questionnaire records after the questionnaires were destroyed and the cluster numbers scrambled. Further details of dried blood samples collection storage and type of tests used, HIV testing coverage, questionnaires, sampling frame, sample allocation, sample selection, and selection of probability and sampling weights have been described and reported elsewhere [24].

### Statistical analysis

Analysis for this paper was restricted to men and women who were both interviewed and HIV tested. Analysis of association with HIV infection, undiagnosed HIV infection and ever been HIV tested was restricted to sexually active participants. The SLDHS 2008 did not include a question on perceived HIV status. Those who reported a previous voluntary HIV test, and were found HIV positive in this survey, were excluded from certain analyses because it was assumed they would be aware of their HIV infection, and their attitudes and knowledge of HIV and their recent sexual behaviour may have changed as a result of diagnosis. They were excluded from all analyses of knowledge and attitudes towards HIV and from the analyses examining the association between HIV infection and recent sexual behaviour and attitudes, thereby providing data about undiagnosed HIV infection. Whilst the analysis of HIV testing was first performed on the whole sample, the analysis to see how testing was associated with recent behaviour and attitudes was repeated excluding those considered aware of their infection to see if associations were appreciably altered.

Weights were calculated that account for the differential sampling probability of each household and also that reflect the differential non-response (to interview and to HIV testing) by gender. Specifically the weights account for the over-sampling of smaller strata, and the lower probability of selection for a household in a larger enumeration area, further details are available elsewhere [24]. To compare sample characteristics by gender the complex survey equivalent chi-squared test was used. To measure the association of behavioural, demographic and risk perception factors with HIV infection and reporting a voluntary HIV test logistic regression was used. To identify the key factors independently associated with HIV infection, undiagnosed HIV Infection and HIV testing, we used model selection based on a ‘manually’ conducted backwards stepwise procedure, with a threshold for inclusion and exclusion of p = 0.1 and including in the process only factors initially found significant in the univariate analysis (p<0.1).

To test the robustness of our two final multiple regression models, for HIV infection and having ever had a voluntary HIV test, we fitted skewed logistic regression models. Standard logistic regression assumes that individuals with a probability of the dependent variable of 0.5 are most sensitive to changes in the independent variables, whereas skewed logistic regression allows the greatest change for individuals whose probability is lower or higher than 0.5 [27]. Skewed logistic regression is therefore more flexible.

For each of the three outcome measures we tested gender interactions with each of the socio-demographic, health, service use, HIV knowledge and attitude factors that are listed in the tables and found little evidence of gender interaction (2 significant interactions in 27 tested, p<0.05) and therefore all results are presented for men and women combined. We performed all analyses using the survey analysis functions of Stata (version SE 13; Stata Corp., College Station, TX, USA), accounting for the clustering and weighting of the sample. The percentages and odds ratios presented are weighted, frequency counts are unweighted. Skewed logistic regression was performed using the function ‘scobit’. A 5% significance level was used, unless otherwise stated.

### Ethical approval and data source

The data were obtained for analysis from DHS Data Archive [28] and were supplied without personal identifiers. This secondary analysis research study was approved by the Ethical and Sanitation Committee of Sierra Leone and University College Hospital Ethics Committee judged that formal ethical approval was unnecessary.

## Results

### Characteristics of survey participants

A total of 7495 participants were asked to complete an interview and also offered an HIV test and 6475 participants (3009 men and 3466 women) accepted both components and hence are included in all analyses (Table 1) giving a response rate of 86% (see the SLDHS 2008 Report for further details [24]).

**Table 1:** Sample Characteristics by Gender.

Variable		Male, %(n)	Female, %(n)	p-value for gender diff
Total		N = 3009	N = 3466	
Socio-demographic factors				
Age	15–19 yrs	16.1(502)	15.3(570)	NA
	20–24 yrs	12.1(369)	16.2(566)	
	25–29 yrs	13.5(399)	22.4(744)	
	30–34 yrs	11.6(353)	13.7(487)	
	35–39 yrs	16.5(486)	16.5(538)	
	40–44 yrs	10.2(307)	8.9(318)	
	45–49 yrs	10.1(303)	7.0(243)	
	50–59 yrs^1^	5.5(161)	NA	
Religion	Christian	21.3(737)	23.0(914)	P = 0.143
	Muslim or other	78.7(2256)	77.0(2519)	
Marital Status	Married	58.6(1730)	67.1(2252)	P<0.001
	Unmarried	38.2(1174)	26.6(987)	
	Widowed/Separated/Divorced	3.2(105)	6.3(227)	
Educational level or number of years formal education	No education	49.3(1415)	67.2(2182)	P<0.001
	Primary	14.8(459)	13.8(499)	
	Secondary	31.2(954)	16.9(662)	
	Higher	4.6(155)	2.1(88)	
Region	Eastern	18.5(755)	17.4(809)	P = 0.040
	Northern	38.7(819)	41.6(1018)	
	Southern	21.8(828)	21.3(921)	
	Western	21.0(607)	19.8(718)	
Residence	Urban	37.3(1247)	35.0(1427)	P = 0.025
	Rural	62.7(1762)	65.0(2039)	
Ever sex	Yes	88.3 (2646)	94.7(3236)	P<0.001
Age at first sex	14&under	8.8(239)	27.2(853)	P<0.001
	15–16	22.4(600)	39.7(1296)	
	17–18	28.0(731)	20.7(671)	
	19&older	40.8(1076)	12.5(415)	
HIV status	HIV +	1.2(32)	1.7(64)	P = 0.068
Ever tested for HIV	Yes	9.1(239)	12.9(454)	P = 0.001
If tested, got result	Yes	83.0(195)	75.2(335)	P = 0.080

^1^Women aged 50–59 years were not eligible for the survey

The majority of the sample were Muslim (78%), and most participants (64%) lived in rural areas. More than half of the sample reported no formal education; illiteracy was significantly higher in women than men (p<0.001). Men were slightly older than women (mean age 32.9 years and 29.3 years respectively, p<0.001). Twice as many women as men reported their first sexual intercourse at 16 years old or younger (p<0.001). In the sample 59% of men were married, 38% unmarried and 3% widowed, separated or divorced compared to 67%, 27% and 6% of women respectively (p<0.001). Most (78%, 71/91) of those who were HIV positive had never had a voluntary HIV test; 86% were sexually active in the last 12 months and 96% of those did not use a condom at last sexual intercourse.

More women than men had ever had a voluntary HIV test (p = 0.001). 7.5% of women had a test as part of antenatal care. Antenatal care was the source of testing for 60% of all women that had an HIV test and 81% among women who tested and who had given a birth in the last 5 years. There is some evidence that the HIV prevalence is higher in women than men (p = 0.068) in this analysis based on all participants whether sexually active or not.

### Level of knowledge and attitudes towards HIV

Only 43% of men and 24% of women (p<0.001) knew where to get condoms (Table 2). Almost all (93%) men, but only 61% of women could get a condom if they wanted (p<0.001). Around 38% of both men and women knew a place for HIV testing. Although 82% of men and 69% of women had heard of HIV/AIDS (p<0.001) and slightly more had heard of sexually transmitted infections, awareness of antiretroviral treatments (ART) was much lower at 35% of men and 27% of women. Around three fifths of men and women knew that HIV could be transmitted to a child through pregnancy, delivery or breastfeeding but only 39% of women and 29% of men (p<0.001) had heard of drugs to prevent mother to child transmission. Around three quarters of men and three fifths of women knew the risk of acquiring HIV could be reduced through being faithful, use of condoms and abstaining from sex (p<0.001 in each case). Less than half of male respondents and around half of female respondents were unsure or thought that HIV could be transmitted from mosquito bites, by witchcraft and by sharing food with an HIV infected person (p<0.001 in each case). Almost half of women and a third of men did not know or thought it impossible that a healthy looking person could be HIV positive (p<0.001), and similar proportions of men and women were unsure or would not care for an HIV positive person (p<0.001). Only 39% of men and 20% of women (p<0.001) would buy vegetables from an HIV infected vendor. The vast majority of participants (94% of men and women) did not know an HIV positive person.

**Table 2:** Level of knowledge & attitudes towards HIV by gender^1^.

Variable		Male, %(n)	Female, %(n)	p-value
**Health and service use-related factors**				
Do you know where to get condoms?	No	56.8 (1619)	75.7 (2534)	P<0.001
	Yes	43.2 (1344)	24.3 (912)	
Can you get condoms if you wanted?	No	7.2 (88)	39.1 (330)	P<0.001
	Yes	92.8 (1235)	60.9 (523)	
Do you know where a person can be tested for HIV?	No	61.8 (1508)	61.4 (1469)	P = 0.812
	Yes	38.2 (976)	38.6 (997)	
HIV knowledge and attitudes				
^2^Have you ever heard of STD	No	12.0 (346)	21.4 (655)	P<0.001
	Yes	88.0 (2645)	78.6 (2764)	
Have you ever heard of AIDS	No	17.6 (512)	31.1 (953)	P<0.001
	Yes	82.4 (2490)	68.9 (2484)	
Have you ever heard of ARV	No	39.7 (1017)	46.5 (1175)	P<0.001
	Yes	34.6 (817)	26.7 (651)	
	Don’t know	25.7 (624)	26.8 (650)	
^3^The risk of acquiring HIV is reduced through being faithful	No	9.3 (235)	12.4 (321)	P<0.001
	Yes	79.9 (1977)	68.0 (1657)	
	Don’t know	10.8 (273)	19.6 (499)	
^4^The risk of acquiring HIV is reduced through condom use	No	11.9 (296)	12.3 (330)	P<0.001
	Yes	74.7 (1846)	61.7 (1505)	
	Don’t know	13.4 (334)	25.0 (642)	
^5^The risk of acquiring HIV is reduced by abstaining from sex	No	17.8 (440)	21.1 (538)	P<0.001
	Yes	69.2 (1722)	56.6 (13.96)	
	Don’t know	13.0 (328)	22.3 (550)	
^6^HIV can be acquired from a mosquito bite	No	55.0 (1314)	44.8 (1123)	P<0.001
	Yes	27.0 (701)	26.2 (627)	
	Don’t know	18.0 (469)	29.0 (725)	
HIV can be acquired because of witchcraft	No	70.2 (1730)	57.7 (1498)	P<0.001
	Yes	7.9 (197)	6.9 (157)	
	Don’t know	21.9 (552)	35.4 (813)	
HIV can be acquired by sharing food	No	58.3 (1455)	50.8 (1323)	P<0.001
	Yes	24.4 (584)	22.5 (506)	
	Don’t know	17.3 (443)	26.7 (648)	
A healthy looking person can have HIV	No	12.6 (321)	20.4 (473)	P<0.001
	Yes	69.0 (1695)	52.6 (1320)	
	Don’t know	18.4 (445)	27.0 (656)	
Know person with HIV	No	94.3 (2304)	93.8 (2315)	P = 0.523
	Yes	5.7 (151)	6.2 (153)	
Will take care of person with HIV	No	20.6 (479)	41.0 (945)	P<0.001
	Yes	73.2 (1894)	48.8 (1281)	
	Don’t know	6.2 (116)	10.2 (255)	
Would you by vegetables from a vendor with HIV?	No	56.8 (1394)	75.0 (1809)	P<0.001
	Yes	38.9 (987)	20.0 (536)	
	Don’t know	4.3 (108)	5.0 (135)	
Can HIV infected person be allowed to teach?	No	37.0 (917)	59.8 (1426)	P<0.001
	Yes	52.2 (1330)	30.8 (807)	
	Don’t know	10.8 (241)	9.4 (248)	
Can HIV be transmitted by breastfeeding?	No	14.1 (342)	10.9 (264)	P = 0.075
	Yes	60.3 (1473)	63.7 (1594)	
	Don’t know	25.6 (673)	25.4 (622)	
Can HIV be transmitted by delivery	No	12.0 (323)	12.1 (302)	P = 0.765
	Yes	60.0 (1438)	58.6 (1474)	
	Don’t know	28.0 (729)	29.3 (706)	
Can HIV be transmitted by pregnancy	No	8.9 (245)	9.6 (241)	P = 0.509
	Yes	66.1 (1587)	63.9 (1624)	
	Don’t know	25.0 (657)	26.5 (617)	
Heard of drugs that can be given to AIDS infected women to reduce the risk of mother-to-child transmission?	No	27.4 (530)	33.4 (623)	P<0.001
	Yes	39.3 (680)	29.1 (510)	
	Don’t know	33.3 (600)	37.5 (669)	

^1^HIV infected & previously voluntarily tested excluded (n = 20).

^2^Apart from AIDS, have you heard about other infections that can be transmitted through sexual contact?

^3^Can people reduce their chance of getting the AIDS virus by having just one uninfected sex partner who has no other sex partners?

^4^Can people reduce their chance of getting the AIDS virus by using a condom every time they have sex?

^5^Can people reduce their chance of getting the AIDS virus by not having sexual intercourse at all?

^6^Can people get the AIDS virus from mosquito bites?

### Associations with HIV infection

After restricting analysis to sexually active participants, the HIV prevalence was 1.3% in men and 1.8% in women. In univariate analysis (Table 3) HIV infection was associated with marital status (p = 0.018), separated, divorced or widowed individuals have a prevalence three times higher than those married. HIV infection was associated with age at first sex (p = 0.026), those who had their first sex at 17–18 years old were more likely to be HIV infected than those who first had sex at a younger or an older age. Urban populations had a higher HIV prevalence than rural communities (p<0.001), and those residing in the Western province were more likely to be HIV positive than those living elsewhere (p = 0.003). After model selection from among the factors in Table 3 urban residence was found to be significantly associated with HIV infection and there was some evidence also of association with marital status (p = 0.078) and age at first sex (p = 0.055).

**Table 3:** Association of ddemographic and early life factors with HIV Infection in sexually active participants.

Variable		% HIV+ (n)	Odds ratio(95% CI)	p-value	Adjusted OR(95% CI)	p-value multivariate model
Total		1.5 (96)				
Socio-demographic factors						
Age	15–24	1.3 (23)	1	*P = 0*.*265*		
	25–34	2.1 (40)	1.56 (0.89–2.73)			
	35–44	1.4 (21)	1.06 (0.54–2.08)			
	45–59	1.3 (10)	0.97 (0.41–2.32)			
Gender	Male	1.3 (32)	1	*P = 0*.*163*		
	Female	1.8 (62)	1.40(0.88–2.11)			
Religion	Christian	1.6 (24)	1	*P = 0*.*852*		
	Muslim	1.6 (68)	0.95 (0.55–1.65)			
Marital Status	Married	1.3 (47)	1	***P = 0*.*018***	1	*P = 0*.*078*
	Unmarried	2.0 (32)	1.59 (0.92–2.75)		1.19 (0.70–2.02)	
	Separated, divorced, widowed	3.7 (15)	3.02 (1.33–6.84)		2.58 (1.14–5.85)	
Educational level	No education	1.4 (45)	1	*P = 0*.*385*		
	Primary	1.8 (14)	1.33 (0.67–2.62)			
	Secondary & Higher	2.0 (34)	1.51 (0.84–2.70)			
Region	Eastern	1.4(22)	1	***P = 0*.*003***		
	Northern	1.3(20)	0.98 (0.48–2.03)			
	Southern	0.8(15)	0.60 (0.25–1.46)			
	Western	3.1(37)	2.35 (1.21–4.58)			
Residence	Urban	2.7(63)	1	***P<0*.*001***	1	***P = 0*.*002***
	Rural	1.0(31)	0.37 (0.21–0.65)		0.41 (0.23–0.72)	
Age at first sex (years)	14 & under	1.5 (18)	1	***P = 0*.*026***	1	*P = 0*.*055*
	15–16	1.3 (25)	0.83 (0.42–1.65)		0.81 (0.41–1.61)	
	17–18	2.7 (37)	1.75 (0.88–3.46)		1.58 (0.79–3.17)	
	19 & older	1.0 (14)	0.63 (0.24–1.67)		0.63 (0.23–1.69)	

### Associations with undiagnosed HIV infection

To examine undiagnosed HIV infection all HIV positive individuals who reported they had ever taken a voluntary HIV test were excluded (n = 20). Univariate analysis of undiagnosed HIV infection did not find any association with knowledge, behaviour and attitude factors (Table 4).

**Table 4:** Association of recent behaviour and attitudes with undiagnosed HIV infection in sexually active participants^1^.

Variable		% HIV+ (n)	Odds ratio(95% CI)	p-value
Total		1.2 (76)		
**Recent behaviour**				
No. of sex partners, last year	no sex	0.9 (8)	1	P = 0.652
	1 partner	1.3 (52)	1.43(0.54–3.78)	
	2+ partners	1.6 (10)	1.70(0.55–5.20)	
Use condom last time had sex	No	1.4 (59)	1	P = 0.415
	Yes	0.8 (2)	0.55(0.13–2.32)	
**HIV knowledge and attitudes**				
Have you ever heard of AIDS	No	1.3 (15)	1	P = 0.844
	Yes	1.3 (59)	0.93(0.45–1.9)	
HIV risk is reduced through being faithful	No	0.8 (3)	1	P = 0.545
	Yes	1.3 (46)	1.67(0.49–5.61)	
	Don’t know	1.6 (10)	2.15(0.54–8.62)	
HIV risk is reduced through condom use	No	0.8 (5)	1	P = 0.529
	Yes	1.3 (41)	1.69(0.59–4.86)	
	Don’t know	1.5 (13)	2.00(0.59–6.73)	
Healthy looking person can have HIV	No	1.0 (5)	1	P = 0.789
	Yes	1.2 (39)	1.25(0.40–3.92)	
	Don’t know	1.4 (13)	1.49(0.43–5.13)	
HIV risk is reduced by not having sex at all	Yes	1.5 (15)	1	P = 0.471
	No	1.1 (32)	0.73(0.38–1.39)	
	Don’t know	1.5 (12)	1.03(0.42–2.49)	

^1^HIV infected & previously voluntarily tested excluded (n = 20).

### Associations with having a voluntary HIV test

Twelve per cent of the sample had ever taken a voluntary test for HIV, more commonly reported by women than men (p = 0.001). Around 80% of those tested got their result. In univariate analysis ever having an HIV test (Table 5) was associated with being 25–34 years old, urban residence, living in the Western province, Christian religion, female with a child<5 years old, having secondary or higher education and having had first sex at 17–18 years of age. Using a condom at last sex, knowing that HIV can be reduced by abstinence, being faithful or through condom use, and knowing that a healthy looking person can have HIV were all associated with ever taking a voluntary HIV test. After adjustment, most of the variables that were significant in univariate analysis, remained significant (Table 5). Participants who were living in urban areas or in Southern or Western regions, female with or without children under 5 years of age, of secondary/higher education, who had first sex at 17 years of age or older, used condom at last sex, and know that HIV can be reduced by using condom were more likely to have been tested. Univariate and multivariate analysis were repeated excluding those who were found HIV positive in this survey and who reported a previous voluntary HIV test, and the results were similar [data not shown].

**Table 5:** Associations with ever having a voluntary HIV test in sexually active participants.

Variable		% HIV tested (n)	Odds ratio (95% CI)	p-value	Adjusted OR (95% CI)	p-value multivariate model
Total		12.0 (680)				
**Socio-demographic factors**						
Age	15–24	12.4 (174)	1	***P<0*.*001***	1	***P = 0*.*013***
	25–34	13.9 (263)	1.14 (0.90–1.46)		1.43(1.04–1.97)	
	35–44	11.9 (185)	0.95 (0.74–1.22)		1.69 (1.21–2.37)	
	45–59	6.8 (58)	0.52 (0.37–0.73)		1.21 (0.75–1.95)	
Gender	Male	9.8 (230)	1	***P<0*.*001***		***P<0*.*001***
	Female with child< 5yrs	16.5 (330)	1.81 (1.42–2.30)		6.49 (4.65–9.06)	
	Female with no child or child> = 5	9.0 (120)	0.90 (0.68–1.20)		1.74(1.26–2.40)	
Religion	Christian	19.6 (264)	1	***P<0*.*001***		
	Muslim	9.8 (413)	0.44 (0.35–0.56)			
Marital Status	Married	11.4 (428)	1	*P = 0*.*201*		
	Unmarried	13.6 (212)	1.23 (0.98–1.55)			
	Separated, divorced, widowed	12.3 (40)	1.09 (0.63–1.63)			
Educational level	No education	6.8 (223)	1	***P<0*.*001***	1	***P<0*.*001***
	Primary	13.6 (90)	2.15 (1.64–2.82)		1.77 (1.27–2.47)	
	Secondary & Higher	23.1 (359)	4.13 (3.2–5.32)		3.01 (2.16–4.19)	
Region	Eastern	7.9 (112)	1	***P<0*.*001***	1	***P = 0*.*008***
	Northern	7.9 (129)	0.96(0.65–1.44)		1.20 (0.80–1.82)	
	Southern	10.6 (132)	1.40(0.85–2.30)		1.85 (1.08–3.15)	
	Western	25.0 (307)	3.90(2.73–5.57)		1.90 (1.30–2.78)	
Residence	Urban	21.1 (454)	1	***P<0*.*001***	1	***P = 0*.*017***
	Rural	6.7 (226)	0.27 (0.20–0.36)		0.62 (0.42–0.92)	
Age at first sex	14&under	9.1 (102)	1	***P<0*.*001***	1	***P = 0*.*006***
	15–16	10.8 (205)	1.20 (0.91–1.60)		1.38 (0.98–1.95)	
	17–18	16.1 (207)	1.91 (1.43–2.56)		1.88 (1.29–2.74)	
	19&older	11.8 (166)	1.33 (1.01–1.76)		1.76 (1.19–2.61)	
**Recent behaviour**						
Last time had sex	In last 12 months	12.1 (549)	1	*P = 0*.*410*		
	>12 months	11.0 (101)	0.89(0.68–1.17)			
Use condom last time had sex	No	11.5 (486)	1	***P<0*.*001***	1	***P = 0*.*030***
	Yes	23.5 (58)	2.37(1.62–3.46)		1.60 (1.05–2.43)	
No. of sex partners, last year	no sex	11.0 (101)	1	*P = 0*.*343*		
	1 sex partner	12.4 (480)	1.15 (0.87–1.51)			
	2 + sex partners	10.5 (58)	0.95 (0.65–1.40)			
**HIV knowledge and attitudes**						
Have you ever heard of AIDS	No	0	-			
	Yes	14.7 (680)	-			
HIV risk is reduced through being faithful	No	14.6 (73)	1	***P = 0*.*006***		
	Yes	16.2 (563)	1.13 (0.82–1.57)			
	Don’t know	7.5 (40)	0.47 (0.27–0.83)			
HIV risk is reduced through condom use	No	12.7 (77)	1	***P<0*.*001***	1	***P = 0*.*019***
	Yes	16.9 (535)	1.40 (1.03–1.92)		1.33 (0.93–1.91)	
	Don’t know	8.0 (65)	0.60 (0.38–0.96)		0.74 (0.43–1.26)	
Healthy looking person can have HIV	No	12.4 (97)	1	***P<0*.*001***		
	Yes	17.7 (495)	1.52 (1.14–2.02)			
	Don’t know	8.3 (77)	0.64 (0.42–0.99)			
HIV risk is reduced by not having sex at all	Yes	17.6 (156)	1	***P<0*.*001***		
	No	15.9 (470)	0.88 (0.69–1.13)			
	Don’t know	7.4(54)	0.37 (0.23–0.59)			

### Sensitivity analysis

For each of our two final multiple regression models, for HIV infection and having ever had a voluntary HIV test, we also fitted the corresponding model using skewed logistic regression. For both outcome variables we compared the p-values and coefficients from the standard and skewed logistic regression for each explanatory term in the models and these were near identical with near identical 95% confidence intervals (results are not reported). Consequently we believe the findings we report from our original multiple logistic regression models are robust to allowing the more general form of skewed logistic regression.

## Discussion

The SLDHS 2008 estimated the national HIV prevalence in the general population of Sierra Leone aged 15–59 years to be 1.5%, and slightly higher in women than men. Most (79%) HIV infected adults were unaware of their HIV infection; the vast majority (90%) reported being sexually active in the last 12 months and most (80%) did not use condom at last sexual intercourse. Only a third of women and men aged 15–59 years old knew where to obtain an HIV test. One tenth of the sexually active population, and significantly fewer men than women, had ever tested for HIV and only 5% had tested in the last 12 months.

Across sub-Saharan Africa the DHS surveys provide broadly similar findings concerning the HIV undiagnosed fraction and associations with testing. While seropositive individuals are more likely to have ever been tested for HIV, most of those living with HIV remain undiagnosed and have no way of knowing their HIV status because they have never been tested [18]. In each of the 29 countries surveyed, higher percentages of urban than rural residents report ever having been tested for HIV. Older adults have more lifetime exposure to the possibility of being tested. HIV testing uptake tends to be higher among those who attended secondary education compared with those with no education or with primary education only. Never-married women and men report lower uptake of HIV testing. Higher percentages of sexually active respondents report ever testing and testing in the past 12 months than respondents who have never had sex [18]. For a regional comparison we note that relative to Sierra Leone the 2008 DHS surveys in Ghana and Nigeria indicated that more (70% and 49% respectively) of women and men aged 15–49 years knew where to obtain an HIV test, somewhat more (15% in both countries) had ever tested and received the results of the test and a similar proportion (5.5% and 6.6% respectively) had tested and received the results in the last 12 months [29, 30].

Whilst we have no data concerning the impact of diagnosis or subsequent prevention activities on transmission within Sierra Leone other studies in sub-Saharan Africa have shown that behavioural interventions with PLWHAs based on increasing knowledge and awareness of HIV transmission factors, and offering prevention and partner counselling, have been associated with reduced HIV transmission risk [1,31,32]. In Uganda, Kenya and Malawi, knowledge of HIV status was associated with a three-fold increase in condom use in a nationally representative survey [1,4], and in Uganda, among patients on ART, prevention counselling and partner voluntary counselling and testing (VCT) was associated with a 70% reduction in sexual risk behaviour and 98% reduction in HIV transmission risk [32].

There is an urgent need to improve the HIV testing uptake in Sierra Leone, a necessary first step to access treatment, change behaviour and positive prevention in those infected, and an opportunity also to promote prevention to those uninfected. The overall testing coverage in 2008 remained far below Sierra Leone’s national goal of 100% testing of all adolescents and adults [33]. HIV testing is higher among women who had given a birth in the last 5 years, compared to those who did not, and compared to men, and the vast majority (81%) of these women who reported testing stated this was as part of antenatal care. This confirms the importance of ANC services for HIV testing in women of reproductive age. Nevertheless only 16% of women who had given birth in the last 5 years have been tested for HIV. The National Guidelines for HTC in Sierra Leone promote a varied approach including mass HIV testing campaigns, antenatal care clinics, mobile clinics, client-initiated and provider-initiated [33]. Our findings support these guidelines and also suggest a need for special efforts to further promote HIV testing as part of antenatal care and to bring HIV testing to men and to women who are not using ANC services. Whilst most demographic subgroups with higher HIV prevalence also have higher testing, and vice-versa, some do not and these should be targeted for HIV testing initiatives. This suggests targeting those unmarried (particularly those separated, divorced or widowed), or without education, or aged over 45, and possible consideration of how differences in testing by religion can be addressed.

We have seen that HIV knowledge is low among the general population of Sierra Leone and lower in women than men. We found that only just over 30% of the general population have heard of ART and knew of a place where to get a condom. Increasing promotion of, and access to, condoms is fundamental to HIV prevention efforts. While provision of information is not in itself enough to change behaviours it is a fundamental component of HIV prevention. Raising awareness of ART could help encourage people to test for HIV, especially antenatally, reduce stigma, and increase use of ART to prevent mother-to-child transmission.

There are some limitations to this study that should be noted. The SLDHS is a household survey, and therefore excludes non-household population groups, such as those living on the street or in institutions (e.g. prisons, boarding schools, military barracks, refugee camps, and brothels). These non-household populations may have higher HIV prevalence than the household population. Whilst the response rates to interview and HIV testing were comparatively high, we cannot exclude the possibility that non-responders may differ from responders in key outcomes such as HIV infection. The missing data may have caused some bias in prevalence estimates. The measures of association with HIV infection and testing we present, and which are the focus of our work, are perhaps less susceptible to bias. The analysis is based on self-reported behaviours, so men and women may misreport these behaviours, for example because of social desirability bias. The analysis is based on cross-sectional data, so the reported associations may not imply causality. The SLDHS 2008 did not collect information on knowledge of HIV status at the time of survey completion. We assumed that those individuals who tested HIV positive in this survey and who reported a previous voluntary HIV test would be aware of their positive status and so we excluded them from certain analyses because of the likely change in their behaviour and attitudes from diagnosis. However it may be that they tested HIV negative at their last voluntary test, and in this case our analysis may underestimate undiagnosed infection in Sierra Leone.

The SLDHS was repeated in 2013 and preliminary findings are available [34], but these do not include data concerning either HIV infection or HIV testing. In due course it will however be possible to repeat these analyses and investigate the change over time.

The HIV epidemic is currently stable in Sierra Leone. However the risk of an escalation remains as health literacy around HIV and HIV prevention is low and 78% of those infected are unaware of their HIV infection and therefore unable to either access therapy or modify behaviour. Many African countries have low HIV testing coverage and so are missing the opportunity for positive prevention. In common with several other sub-Saharan African countries [18], Sierra Leone needs to continue the rapid expansion of HIV testing, access to ART treatment and condoms for people living with HIV.
